# Pharmacological evidence that D-aspartate activates a current distinct from ionotropic glutamate receptor currents in *Aplysia californica*

**DOI:** 10.1002/brb3.60

**Published:** 2012-07

**Authors:** Stephen L Carlson, Andrew T Kempsell, Lynne A Fieber

**Affiliations:** Division of Marine Biology and Fisheries, Rosenstiel School of Marine and Atmospheric Science, University of Miami4600 Rickenbacker Cswy., Miami, Florida 33149

**Keywords:** APV, buccal ganglion, coagonist, electrophysiology, mollusk, NMDA

## Abstract

D-Aspartate (D-Asp) activates a nonspecific cation current of unknown identity independent of L-glutamate (L-Glu) in neurons of *Aplysia californica*. Whole-cell voltage clamp studies were conducted using primary cultures of *Aplysia* buccal S cluster (BSC) neurons to characterize these receptor channels pharmacologically. The N-methyl-D-aspartate receptor (NMDAR) coagonist glycine potentiated D-Asp currents only at −30 mV, while D-serine did not potentiate D-Asp currents at any amplitude. Portions of D-Asp currents were blocked by the L-Glu antagonists kynurenate, DL-2-amino-5-phosphonopentanoic acid (APV), (2S,3R)-1-(phenanthren-2-carbonyl)piperazine-2,3-dicarboxylic acid (PPDA), and 1,3-dihydro-5-[3-[4-(phenylmethyl)-1–2H-benzimidazol-2-one (TCS46b), suggesting that L-Glu channels, particularly NMDAR-like channels, may partially contribute to D-Asp whole-cell currents. In contrast, L-Glu currents were unaffected by APV, and showed greater block by kynurenate, suggesting that D-Asp and L-Glu act, in part, at different sites. The excitatory amino acid transport blocker DL-threo-b-Benzyloxyaspartic acid (TBOA) blocked a fraction of D-Asp currents, suggesting that currents associated with these transporters also contribute. Non-NMDA L-GluR antagonists that preferentially block alpha-amino-3-hydroxyl-5-methyl-4-isoxazole-propionic acid (AMPA)/kainate receptors significantly increased D-Asp currents, suggesting a possible allosteric potentiating effect of these antagonists on D-Asp receptors. L-Glu-induced currents were significantly reduced in the presence of bath-applied D-Asp, whereas bath-applied L-Glu had no effect on D-Asp-induced currents. The mixed effects of these agents on D-Asp-induced currents in *Aplysia* illustrate that the underlying channels are not uniformly characteristic of any known agonist associated channel type.

## Introduction

D-Aspartate (D-Asp) is present at multiple receptor sites in the *Aplysia* nervous system (Zhao and Liu 2001), and activates a nonspecific cation channel, impermeable to Ca^2+^, in *Aplysia* neurons (Carlson and Fieber 2011). In our prior studies, 25% of buccal S cluster neurons and 48% of pleural ventrocaudal neurons had D-Asp-elicited whole-cell currents but lacked L-glutamate (L-Glu) induced responses (Fieber et al. 2010; Carlson and Fieber 2011). Additionally, D-Asp activated currents independently of the L-GluR agonists AMPA and NMDA (Carlson and Fieber 2012). These observations suggest D-Asp activates a dedicated D-Asp receptor, expanding the view that D-Asp acts as an alternate agonist at NMDAR channels (Olverman et al. 1988; Kiskin et al. 1990; Huang et al. 2005), but the identity of these non-L-Glu channels activated by D-Asp is not known. Further, it is unknown if D-Asp and L-Glu activate the same receptor channel in those cells responding to both agonists, or if D-Asp receptors, such as NMDAR, possess an obligatory coagonist binding site that must be occupied by glycine (Gly) or D-serine (D-Ser) for full activation of the channel (Johnson and Ascher 1987; Kleckner and Dingledine 1988).

One of the inherent challenges in working with L-Glu receptors is that many neurons express multiple types of receptors, including NMDA, AMPA, and kainate receptors, and that these subtypes can be further subdivided based on variations in subunit composition (Dingledine et al. 1999). In recent decades, however, a number of pharmacological agents have been developed that have facilitated isolation of currents associated with these channels in electrophysiological investigations (Kew and Kemp 2005; Lodge 2009). Indeed, many of the studies investigating the role of L-Glu in synaptic plasticity have relied largely on pharmacological evidence for identification of the receptors being studied (reviewed in Antzoulatos and Byrne 2004). Despite the professed role of D-Asp as an alternate agonist at NMDARs, pharmacological evidence supporting this hypothesis is limited to a single study (Errico et al. 2011).

Errico et al. (2011) investigated electrophysiological responses to supraphysiological levels of D-Asp in 13- to 15-day-old C57BL/6J mice. The authors reported approximately 67% block of D-Asp-induced currents with NVP-AAM077, cis-PPDA, and Ro 25–6981, NMDAR antagonists selective for NR2A, NR2C/D, and NR2B subunits, respectively, approximating the degree of block of NMDA-induced currents in the same cells. When these three antagonists were applied together or when MK-801, a comprehensive NMDAR blocker, was applied, NMDA currents were completely blocked while D-Asp-activated currents were reduced 80%. These results suggested that while D-Asp activated currents in the hippocampus are similar enough to NMDARs currents to be blocked by NMDAR blockers, it also activated a current clearly not due to NMDAR activation.

There is considerable evidence that D-Asp plays a modulatory role at L-Glu-activated receptors. Antagonistic effects of D-Asp have been observed in L-Glu channels in *Aplysia* (Dale and Kandel 1993) and in rat hippocampal neurons and *Xenopus* oocytes expressing AMPARs (Gong et al. 2005). Further, D-Asp slowed the gating kinetics of a squid glutamate receptor, SqGluR (Brown et al. 2007). In none of these models, however, was D-Asp activation of ion channels studied. It is thus unknown whether D-Asp acts in dual roles, both as a modulator of L-GluR channels and as a neurotransmitter at novel receptors.

The purpose of this study was to further elucidate the identity of channels activated by D-Asp. To achieve this, we attempted a pharmacological characterization of the D-Asp-induced current in *Aplysia* neurons, with a focus on antagonists and coagonists of L-Glu receptor channels.

## Materials and Methods

### Cell culture

*Aplysia californica* (∼300–800 g; six to nine months of age and both immature and sexually mature) were obtained from the University of Miami NIH National Resource for Aplysia in Miami, Florida. Primary cultures of BSC cells were prepared as in Fieber (2000) and Carlson and Fieber (2011). Briefly, animals were anesthetized for 1 h in a 1:1 mixture of seawater and 0.366 M MgCl_2_. Buccal ganglia were then dissected out and placed in 5-mL artificial seawater (ASW) consisting of (mM): 417 NaCl, 10 KCl, 10 CaCl_2_ (2 H_2_O), 55 MgCl_2_ (6 H_2_O), 15 HEPES-NaOH, pH 7.6) plus 100 Units/mL penicillin and 100 mg/mL streptomycin, with 18.75 mg dispase (Boehringer Mannheim 10165859001), 5 mg hyaluronidase (Sigma H4272), and 1.5 mg collagenase type XI (Sigma C9407) and shaken at low speed for approximately 24 h at room temperature (∼22°C). BSC cells were then dissociated onto 35 mm diameter polystyrene culture plates (Becton Dickinson, Falcon Lakes, NJ) coated with poly-D-lysine (MP Biomedicals IC15017525). Cells were stored at 17°C until used in experiments up to 48 h later.

### Electrophysiology

Whole-cell voltage clamp and current clamp measurements were made using glass patch electrodes pulled from thick-walled 1.5 mm diameter borosilicate filament glass capillaries using a Flaming/Brown micropipette puller (Sutter Instruments, Novato, CA). Voltage and current data were collected and whole-cell capacitance and series resistance compensations were made using an Axopatch 200B clamp amplifier, with a capacitance compensation range of 1–1000 pF, connected to a PC and Digidata 1200 A/D converter using pClamp software to record data and issue voltage and current commands (Molecular Devices, Sunnyvale, CA). ASW and experimental solutions were flowed onto cells during recording via a 6-bore gravity-fed perfusion system that dispensed solution from a 1 μL, 0.199-mm internal diameter micropipette (Drummond Scientific, Broomall, PA) approximately 100 μM from the cell, capable of changing the local bathing environment around cells within 500 msec. D-Asp and L-Glu (1 mM) were made in ASW from frozen stocks that had been prepared from 0.5 M D-aspartic acid or L-glutamic acid in 0.5 M NaOH. Solutions containing agonist were briefly applied via filament borosilicate glass capillary tubes pulled to a similar shape and tip diameter as the patch electrodes, aimed at and positioned within approximately 30 μM of the cell at a 45° angle to the perfusion pipette that was attached to a picospritzer powered by N_2_ at standardized pressure and duration. Unless otherwise noted, the agonists (i.e., D-Asp or L-Glu) were applied via the picospritzer for 100 msec at a concentration of 1 mM.

### Pharmacology

Due to the desensitizing nature of D-Asp-activated currents (Carlson and Fieber 2011), a pause of 80 sec was observed between each application of agonist. Pharmacological experiments were performed as a three-pulse protocol, with inhibition of current assessed as a proportion of maximal current amplitude. An initial control application of agonist from the picospritzer pipette in flowing ASW was followed by a switch of the bathing solution to one containing blocker. An 80 sec interval before application of a second pulse of agonist allowed the possibility of full block by competitive antagonists. The bathing solution was then switched back to ASW and a third application of agonist was made to study washout of the antagonist. When L-Glu and D-Asp currents were studied in the same cell, the agonist pipette was changed after washout and this protocol was repeated. Agonist order was alternated with each cell studied. For Gly and D-Ser experiments, the agonist pipette was changed from one containing D-Asp to one containing D-Asp + Gly or D-Asp + D-Ser after control currents for D-Asp were recorded. Because the noncompetitive antagonists memantine and MK-801 require channel opening for block to occur, two applications of agonist were made during antagonist exposure before washout, and each application was compared to the control. For analysis, D-Asp and L-Glu current amplitudes in pharmacological agents were normalized to the initial control current. Unless otherwise noted, pharmacological experiments were performed at −30 mV, approximately the resting potential for cultured BSC neurons (Fieber et al. 2010).

Cyclothiazide (CTZ) experiments were performed both under the conditions described above and under conditions designed to investigate block of desensitization. For desensitization experiments, cells were exposed to repeated applications of D-Asp both in ASW and in ASW + CTZ. Three applications of D-Asp were made: an initial, control application (*t*_0_), an application at *t*_10_=*t*_0_+ 10 sec, and a final application at *t*_20_=*t*_0_+ 20 sec. Currents were normalized to the control current for each condition.

### Solutions

Unless noted, all reagents were from Sigma-Aldrich (St. Louis, MO). Control extracellular solution consisted of ASW. Control intracellular solutions contained (mM) 458 KCl, 2.9 CaCl_2_ (2 H_2_O), 2.5 MgCl_2_ (6 H_2_O), 5 Na_2_ATP, 1 EGTA, and 40 HEPES-KOH, pH 7.4.

For pharmacological experiments, competitive and noncompetitive antagonists of L-Glu receptors or Cl^−^ channel blocker were diluted in ASW from frozen stocks. The protocol entailed application of blocker to cells after control records in response to D-Asp or L-Glu were made, then washout of any blocker effects. Blocker concentrations were selected based on prior experiments in *Aplysia* (Dale and Kandel 1993; Armitage and Siegelbaum 1998; Klein et al. 2000; Chitwood et al. 2001; Antzoulatos et al. 2003; Jin and Hawkins 2003; Collado et al. 2007). Stocks of the following drugs were made in water, then diluted in ASW at 1:50 or greater, for their working concentrations shown in parentheses: 4-acetamido-4′-isothiocyanato-2,2′-stilbenedisulfonic acid disodium (SITS; 100 μM), DL-kynurenic acid (kynurenate; 1 mM), DL-2-amino-5-phosphonopentanoic acid (APV; 100 μM), memantine hydrochloride (Tocris, St. Louis, MO; 100 μM), 6-cyano-7-nitroquinoxaline-2,3-dione disodium (CNQX; Tocris; 100 μM), 2,3-dioxo-6-nitro-1,2,3,4-tetrahydrobenzo[f]quinoxaline-7-sulfonamide disodium (NBQX; Tocris; 5 μM), 6,7-dinitroquinoxaline-2,3-dione disodium (DNQX; Tocris; 100 μM), and (R)-(+)-3-amino-1-hydroxypyrrolidin-2-one (HA-966; Tocris; 100 μM). Experiments on blocking effects of HA-966 on currents elicited by D-Asp + D-Ser or D-Asp + Gly were conducted independently of those to assess modulation of D-Asp currents by D-Ser or Gly. Stocks of (5S,10R)-(+)-5-methyl-10,11-dihydro-5H-dibenzo[a,d]cyclohepten-5,10-imine male-ate (MK-801; Tocris; 500 nM) and DL-threo-b-benzyloxyaspartic acid (TBOA; Tocris; 1 mM) were made in ASW. Stocks of (S)-1-(2-amino-2-carboxyethyl)-3-(2-carboxybenzyl)pyrimidine-2,4-dione (UBP 302; Tocris; 50 μM) and CTZ (200 μM) were made in DMSO. The TCS46b (Tocris; 50 μM) stock was made in ethanol. The stock of PPDA (Tocris; 50 μM) was made in 100 mM NaOH (aq.).

### Data analysis

Data are presented as mean ± standard deviation (SD). Differences in current amplitudes with treatments were assessed using Student's paired *t*-tests. Differences in amplitude after desensitization were assessed using two-sample *t*-tests. Analyses were performed using Data Desk software (version 6.2; Data Description, Inc., Ithaca, NY). Differences at *P*≤ 0.05 were accepted as significant.

## Results

### Gly/D-Ser

**Figure 1:**
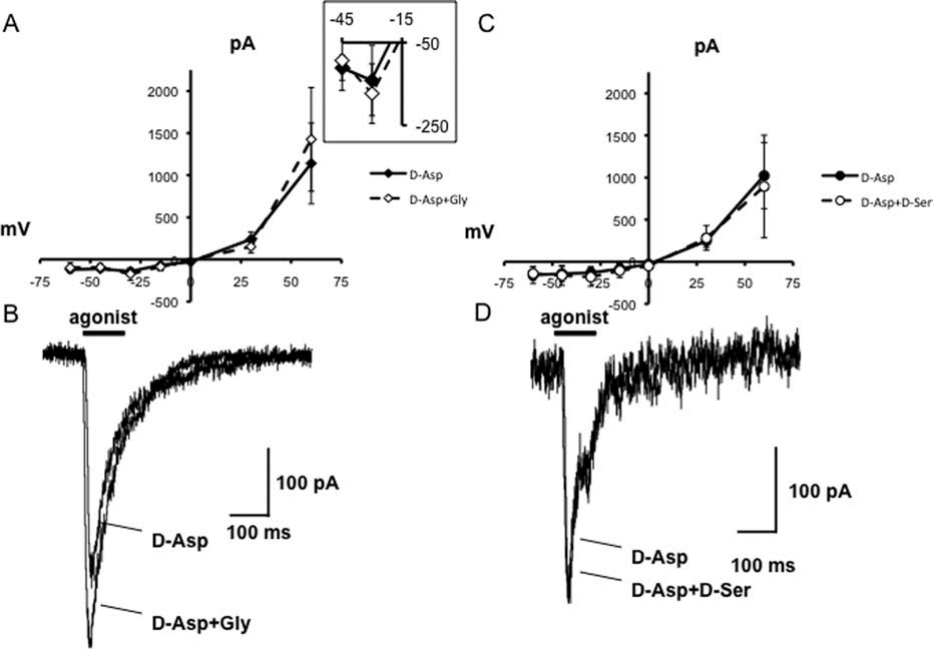
D-Asp responses in BSC neurons in the presence of L-GluR coagonists Gly and D-Ser. (A) Average current–voltage (I–V) relationship ± SD for D-Asp currents (1 mM; 100 msec) with and without Gly added to the pressure ejection pipette (1 mM; *n*= 7). Inset: higher resolution view near −30 mV. (B) Example whole-cell currents in D-Asp and in D-Asp + Gly in the same cell at −30 mV. (C) Average I–V relationship ± SD for D-Asp currents with and without added D-Ser (1 mM; *n*= 7). (D) Example whole-cell currents in D-Asp and in D-Asp + D-Ser in the same cell at −30 mV.

The amplitude of D-Asp currents was compared in the presence and absence of Gly (1 mM) and D-Ser (1 mM), and the current–voltage relationships plotted at voltages from −60 mV to +60 mV (Fig. 1). Current amplitude was significantly greater when D-Asp was coapplied with Gly near the resting potential of BSC cells at −30 mV (Fig. 1A and C; mean increase 24 ± 34%; mean ± SD; *P*≤ 0.05, paired *t*-test, *n*= 14), but not significantly different at any other voltages examined between −60 and +60 mV. There were no significant changes in D-Asp current amplitude in the presence of D-Ser at any of the voltages examined between −60 and +60 mV (Fig. 1B and D). HA-966 (100 μM), a Gly-site antagonist of NMDARs, was tested for block of D-Asp currents both at −30 and 60 mV, to test for voltage-specific effects of Gly at NMDARs that may have contributed to whole currents in response to D-Asp. HA-966 did not block D-Asp currents in the presence or absence of added Gly at −30 and 60 mV (Table 1). When pressure applied to cells in the absence of D-Asp, neither Gly nor D-Ser induced currents in BSC neurons (data not shown).

**Table 1:** Effect of NMDAR glycine-site blocker HA-966 on D-Asp whole cell current amplitude

Agonist	Voltage (mV)	Control current amplitude (mean ± SD)	Current amplitude in HA-966 (mean ± SD)	*n*
D-Asp	−30	−176 ± 75 pA	−173 ± 77 pA	7
D-Asp + Gly	−30	−216 ± 105 pA*	−230 ± 99 pA	8
D-Asp	60	1638 ± 1248 pA	1816 ± 1457 pA	5
D-Asp + Gly	60	2323 ± 1912 pA	2411 ± 1948 pA	5

* Significantly different from D-Asp alone via paired *t*-test, *P*≤ 0.05.

### Pharmacology of D-Asp receptors

**Figure 2:**
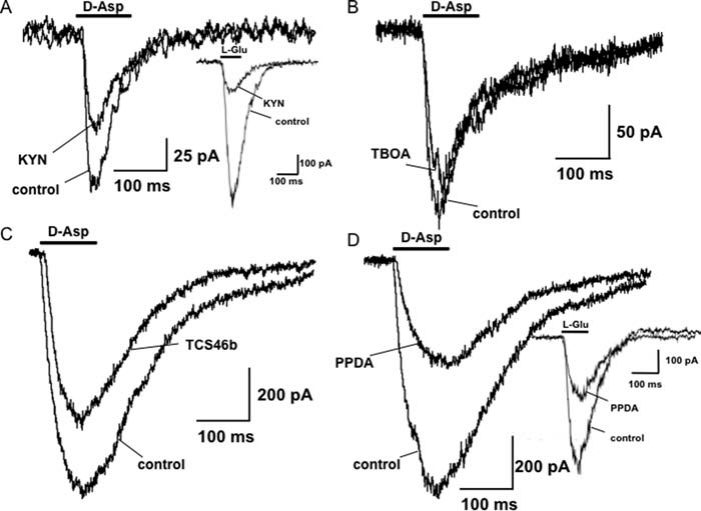
Antagonists of D-Asp currents at −30 mV. (A) Whole-cell currents in response to pressure application of D-Asp (1 mM) in ASW (control) and in ASW plus 1 mM kynurenate (KYN). Inset: whole-cell currents in L-Glu (1 mM) in ASW (control) and in ASW plus 1 mM kynurenate. (B) Whole-cell currents in D-Asp in ASW (control) and in ASW plus 1 mM TBOA. (C) Whole-cell currents in D-Asp in ASW (control) and in ASW plus 50 μM TCS46b. (D) Whole-cell currents in D-Asp in ASW (control) and in ASW plus 50 μM PPDA. Inset: whole-cell currents in L-Glu in ASW (control) and in ASW plus 50 μM PPDA.

**Figure 3:**
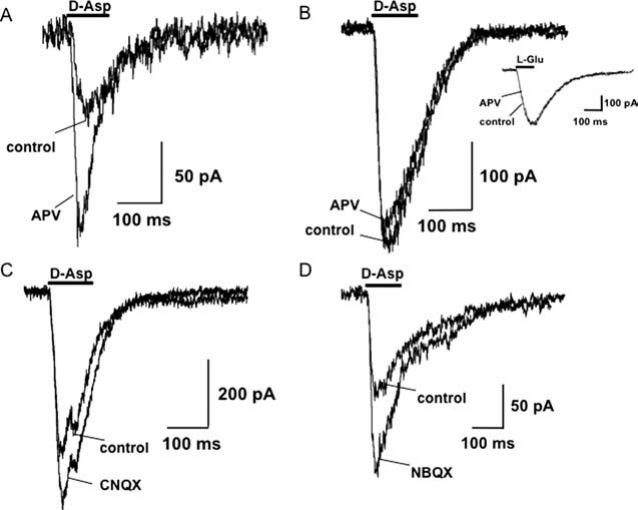
Antagonists of D-Asp currents at −30 mV. (A and B) Currents in D-Asp (1 mM) in ASW (control) and in ASW with 100 μM APV. Inset B: whole-cell currents in L-Glu (1 mM) in ASW (control) and in ASW plus 100 μM APV. (C) Currents in D-Asp in ASW (control) and in ASW plus 100 μM CNQX. (D) Currents in D-Asp in ASW (control) and in ASW plus 5 μM NBQX.

**Figure 4:**
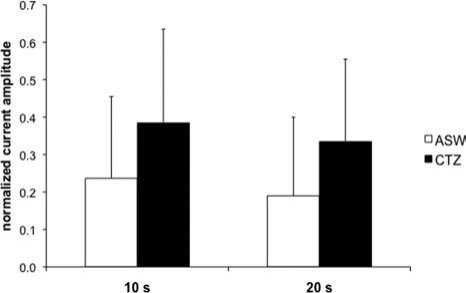
Effects of CTZ on desensitization of D-Asp-induced currents. Control D-Asp-induced currents were elicited in ASW or ASW plus CTZ (200 μM; data not shown), and at 10 and 20 sec later, with the 10 and 20 sec currents plotted as a fraction of their respective control current amplitudes.

**Figure 5:**
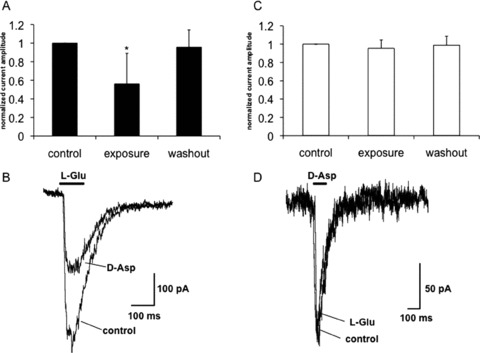
Effects of bath-applied D-Asp and L-Glu on agonist-evoked currents. (A) Summary of effects of 0.5 mM bath-applied D-Asp (exposure) on L-Glu-activated currents (1 mM). *denotes significant difference from control and washout at *P* < 0.05 (Student's paired *t*-test, *n*= 24). (B) Example L-Glu-evoked currents in ASW (control) and in ASW plus D-Asp. (C) Summary of effects of 0.5 mM bath-applied L-Glu on D-Asp-activated currents (1 mM; *n*= 6). (D) Example D-Asp-evoked currents in ASW (control) and in ASW plus L-Glu.

Additional pharmacological data are summarized in Table 2 and Figures 2–5. The Cl^−^ channel blocker SITS (100 μM) was tested for block of D-Asp currents. Since D-Asp currents are not carried by Cl^−^ ions and thus not expected to be blocked by SITS (Carlson and Fieber 2011), this drug served as a control for our experimental conditions. There was no significant difference in current amplitude of D-Asp currents in the presence of SITS (Table 2).

**Table 2:** Summary of effects of antagonists on D-Asp whole-cell currents. Effect on L-Glu currents designated with italics

Antagonist	Target receptor	Percentage of block (−) or potentiation (+)	*N*
SITS (100 μM)	Cl^−^ channels	−1 ± 17%	7
TBOA (1 mM)	EAATs	−10 ± 10%*	8
Kynurenate (1 mM)	Nonselective L-GluRs	−27 ± 19%*	9
		*L-Glu:*−*65 ± 13*%*	*8*
**Role of NMDARs**			
APV (100 μM)	NMDARs	+100 ± 88%*	7
		−22 ± 16%*	15
		*L-Glu:*−*0.8*±*10*%	*8*
PPDA (50 μM)	NMDARs—selective for NR2C/NR2D	−46 ± 22%*	19
		*L-Glu: −46*±*11*%*	*8*
Kyn + APV + PPDA (1 mM + 100 μM + 50 μM)	All L-GluRs	−69 ± 15%*	9
		*L-Glu: −76*±*21*%*	9
TCS46b (50 μM)	NMDARs—selective for NR1A/NR2B	−16 ± 16%*	7
MK-801 (500 nM)	NMDARs	−13 ± 36%	14
Memantine (100 μM)	NMDARs	−6 ± 9%	6
**Role of non-NMDARs**			
CNQX (100 μM)	AMPA/kainate Rs	+30 ± 18%*	8
NBQX (5 μM)	AMPA/kainate Rs	+15 ± 14%*	7
DNQX (100 μM)	Kainate Rs	−22 ± 37%	8
UBP302 (50 μM)	Kainate Rs	+10 ± 23%	6
CTZ (200 μM)	AMPAR desensitization	+3 ± 20%	7

* Significantly different from control recorded in absence of antagonist via paired *t*-test at *P*≤ 0.05.

TBOA (1 mM), a blocker of excitatory amino acid transporters (EAATs), significantly reduced D-Asp currents to a small degree (Fig. 2B; mean decrease 10 ± 10%; *P*≤ 0.05).

D-Asp currents were significantly reduced in amplitude by 27 ± 19% in the presence of kynurenate (1 mM), a general L-Glu receptor antagonist while L-Glu currents in the same cells were uniformly, significantly reduced to a larger extent at 65 ± 13% block (Fig. 2A and Table 2; *P*≤ 0.01, Student's paired *t*-test). Block of both receptors was reversible. The NMDAR antagonist APV (100 μM) had mixed effects, causing a significant, reversible increase in D-Asp current amplitude in 7 of 22 cells examined (Fig. 3A; mean increase of 100 ± 88%; *P* < 0.05), and a significant, reversible decrease in all other cells tested (Fig. 3B; mean block of 22 ± 16%; *P*≤ 0.05). There was no significant difference in D-Asp current amplitude in APV compared to controls when all 22 cells exposed to APV were considered as a single sample. L-Glu currents in the same cells were uniformly unaffected by APV (Fig. 3B, inset; Table 2). PPDA (50 μM), an NMDAR antagonist showing greater preference for vertebrate NR2C and NR2D subunit-containing NMDARs, was the most effective blocker of D-Asp currents observed, at 46 ± 22% block (Fig. 2D and Table 2; *P*≤ 0.01); L-Glu currents in the same cells were blocked to a similar degree on average, although the specific proportion of block of the two receptors in individual cells varied from 31% to 77% with a greater proportion of D-Asp current blocked in some cells, while in other cells more L-Glu current was blocked. PPDA block of both receptors was reversible. Adding the percentage block of L-GluRs by kynurenate (−65 ± 13%) to that by APV (0%) and PPDA (−46 ± 11%) exceeded the observed block of these receptors by a mixture of kynurenate + APV + PPDA (−76 ± 21%). The same was true for D-AspRs, if considering only APV block and not APV-induced potentiation (Table 2).

TCS46b (50 μM), another selective antagonist for subunits NR1A and NR2B of vertebrate NMDARs, caused a slight, significant reduction in D-Asp current amplitude (Fig. 2C and Table 2; mean block of 16 ± 16%; *P*≤ 0.05).

D-Asp currents were not blocked by either MK-801 (500 nM) or memantine (100 μM), potent vertebrate NMDAR antagonists, in either the first exposure to D-Asp or the second application designed to allow the channels to open for the blocker to act (Table 2).

The AMPA/kainate receptor antagonists CNQX (100 μM) and NBQX (5 μM) significantly potentiated D-Asp current amplitude (Fig. 3C and D and Table 2; mean increase of 30 ± 18%, *P*≤ 0.01, and 15 ± 14%, *P*≤ 0.05, respectively), and the effect of each was reversible. DNQX (100 μM) and UBP302 (50 μM), non-NMDAR antagonists with higher specificity to kainate than AMPA receptors, did not block D-Asp currents (Table 2).

D-Asp-induced currents desensitized when agonist was repetitively applied at <40 sec intervals (Carlson and Fieber 2012). CTZ prevents desensitization of AMPARs. Bath applied CTZ (200 μM) had no effect on the first D-Asp currents elicited in BSC neurons (Table 2). CTZ was further tested for its effects on D-Asp currents elicited on a time frame shorter than the normal interval of 80 sec, when desensitization can be observed. The amplitudes of D-Asp currents elicited 10 and 20 sec after a first, control current were normalized to the first current both in normal ASW and in ASW with CTZ (Fig. 4). Compared to their respective controls, there were no significant differences in the amplitudes of D-Asp-induced currents observed at 10 and 20 sec in ASW versus in CTZ (when the second current occurred 10 sec after the control, mean ASW/CTZ = 24 ± 22% of control**/**38 ± 25% of control while at 20 sec = 19 ± 21%**/**33 ± 22%, respectively; *n*= 8).

### Effects of bath-applied agonists on L-Glu and D-Asp receptor currents

D-Asp was tested for its effects on L-Glu-activated currents in BSC neurons. In the presence of bath-applied 0.5 mM D-Asp, L-Glu-induced (1 mM) current amplitude was significantly reduced by 44 ± 33% (Fig. 5A and C; *P*≤ 0.01, *n*= 24). This effect washed out with removal of D-Asp, indicating that the reduction in current amplitude was due to the presence of D-Asp. In contrast, bath-applied L-Glu (0.5 mM) had no effect on amplitude of D-Asp currents (Fig. 5B and D; *n*= 6).

## Discussion

We recently described the D-Asp activated current in *Aplysia* as a nonspecific cation channel that is permeable to Na^+^ and K^+^, but not Ca^2+^ (Carlson and Fieber 2011). Similarly to NMDARs, the currents were blocked by Mg^2+^ at negative voltages. The observation that in many cells the D-Asp-activated currents were not activated by L-Glu suggested activation of unique D-Asp receptors. D-Asp may have multiple receptor targets, some of which overlap with L-Glu. Errico et al. (2011) working in a mouse model suggested that D-Asp acts both at NMDARs and at receptors independent of NMDARs.

Gly and D-Ser are obligatory coagonists at NMDARs (Kleckner and Dingledine 1988) and are not known to be voltage specific. D-Ser had no effect on D-Asp-induced currents, while Gly potentiated them only at −30 mV. It is possible that the potentiating effect of Gly or D-Ser on the portion of D-AspRs that is NMDA-like was diluted within the whole-cell D-Asp current fraction. At the high ionic strength of the solutions used in this study, as much as 100 nM contaminating Gly may have been present even in Gly-free conditions; therefore, we cannot rule out that the NMDA coreceptor site was already occupied in NMDA-like receptors on *Aplysia* neurons (Kleckner and Dingledine 1988). The absence of block by the Gly-site antagonist HA-966 may support the conclusion that, if present, NMDA-like receptors are minor contributors to whole-cell D-Asp-induced currents; however, this result must be interpreted with caution in the absence of studies previously demonstrating the effectiveness of this drug on *Aplysia* NMDA-like receptors. The potentiating effect of Gly observed only at −30 mV may have physiological relevance, however, as this is near the resting potential for cultured BSC neurons (Carlson and Fieber 2012). Thus, Gly potentiation might exert its greatest effect on excitability near the cells’ resting potential, and act as an endogenous mechanism for relieving voltage-sensitive Mg2^+^ block of NMDA-like receptors there.

EAATs are responsible for the reuptake of L-Glu and D-Asp from the extracellular space. These transporters produce an electrogenic current via the uptake of substrate, in which 1 H^+^, 3 Na^+^, and one ligand (e.g., D-Asp or L-Glu) are cotransported into the cell, and one K^+^ countertransported out (Zerangue and Kavanaugh 1996). Additionally, activation of EAATs initiates an uncoupled Cl^-^ conductance in some EAATs (Wadiche et al. 1995). This Cl^-^ conductance would be additive with D-Asp-activated nonspecific cation currents across most of the voltage range (*E*_Cl_=−4.7 mV, while *E*_D-Asp_= 7.7 mV Carlson and Fieber 2012). A number of studies investigating EAATs have utilized D-Asp as an agonist for these transporters (Davies and Johnston 1972; Anderson et al. 1990; Balcar and Li 1992; Apricò et al. 2007), and the EAAT blocker TBOA has been shown to be effective in blocking uptake of L-Glu in a transporter cloned from *Aplysia* (Collado et al. 2007). D-Asp currents in BSC neurons were slightly reduced in TBOA, supporting a small contribution of EAAT activation to D-Asp whole-cell currents.

Kynurenate is a general L-Glu receptor antagonist in vertebrates (Stone 1993), and also was one of the first characterized antagonists of L-Glu-evoked currents in *Aplysia* (Dale and Kandel 1993). A total of 1 mM kynurenate was less potent at blocking D-Asp currents (27%, this study) than L-Glu currents (∼80%, Dale and Kandel 1993; 65%, this study). Block of a small percentage of current by kynurenate suggests that activation of L-Glu receptors partially contributes to D-Asp whole-cell currents, or that D-Asp may activate sharing similar pharmacology to L-Glu receptors.

APV is used extensively in studies investigating synaptic transmission and plasticity associated with learning in *Aplysia* (Glanzman 1994; Lin and Glanzman 1994; Murphy and Glanzman 1997, 1999; Schacher et al. 1997; Conrad et al. 1999; Antonov et al. 2003; Ezzeddine and Glanzman 2003), yet several studies demonstrated no APV sensitivity of putative NMDA-like R responses in *Aplysia* (Antzoulatos and Byrne 2004; Malkinson and Spira 2010) and *Lymnaea* (Moroz et al. 1993); L-Glu currents in BSC cells were unaffected by APV. APV had mixed effects on D-Asp-induced currents in subsets of BSC neurons, blocking 22% of the current in most cells while potentiating D-Asp currents an average of two-fold in a minority of cells. While the slight reduction in current in the presence of APV in 68% of BSC neurons exposed to D-Asp supports, the hypothesis that NMDAR-like channels are partial contributors to D-Asp whole-cell currents, the absence of APV block of L-GluRs in the same cells counters it. In contrast, the potentiating effect of APV on D-Asp currents in some cells may have been mediated via allosteric modulation of the receptor (Kenakin 2004), in which binding of an antagonist may enhance receptor activation by changing the rank order of agonist potency. The portion of D-Asp current in BSC cells not affected by NMDAR antagonists, as well as the potentiating effect of APV, CNQX, and NBQX, suggests novel, and potentially heterogeneous, D-Asp receptors contribute to the whole cell D-Asp response.

PPDA and TCS46b are subunit-specific NMDAR antagonists with PPDA effective in blocking receptors containing NR2C/D subunits (Feng et al. 2004) and TCS46b preferentially blocking receptors containing NR1A/NR2B subunits (Gregory et al. 2000). PPDA was previously demonstrated to inhibit D-Asp currents (Carlson and Fieber 2012) and was the strongest blocker of D-Asp currents observed in this study (∼45%), while TCS46b had minor, but significant, blocking effects. Thus, D-AspR subunits in *Aplysia* may be more similar to mammalian NR2C/D than NR2B subunits. In contrast to our results, Errico et al. (2011) did not observe block of D-Asp receptors that implicated specific NMDAR subunits.

The nonadditive effects of kynurenate, PPDA, and APV block on both L-Glu- and D-Asp-activated currents suggests that these antagonists have some binding sites in common on *Aplysia* L-GluRs and D-AspRs. PPDA block of the two receptors suggests approximately 46% binding of each agonist is pharmacologically similar. The difference in the kynurenate block of the two receptors, however, suggests that an additional approximately 30% of agonist binding is distinct, similar to the findings of Errico et al. (2011).

It is interesting to note that the potent NMDAR channel blockers MK-801 and memantine had no significant effect on D-Asp-induced currents of BSC neurons. While MK-801 partially blocked D-Asp-induced currents in mice CA1 pyramidal neurons (Errico et al. 2011), these drugs do not appear in the literature of *Aplysia* pharmacology, possibly due to a lack of antagonism of NMDA-like receptors in this model. Our results confirm their lack of activity in *Aplysia*.

While the permeability of D-Asp currents is most consistent with AMPA or kainate subtypes of L-Glu-activated receptors (Carlson and Fieber 2011), the pharmacological data suggest that D-Asp activates a channel distinct from these receptors. The AMPA/kainate blockers UBP302 and DNQX had no effect on D-Asp current amplitude. While UBP302 had not been tested in *Aplysia* or other invertebrates, DNQX has been shown to block serotonin-induced facilitation of a putative excitatory AMPAR-mediated response in *Aplysia* siphon motor neurons (Chitwood et al. 2001), L-Glu-induced currents in mechanoafferent neuron B8 (Klein et al. 2000) and at sensorimotor synapses (Dale and Kandel 1993; Armitage and Siegelbaum 1998; Jin and Hawkins 2003), as well as EPSPs and Ca^2+^ transients in pleural sensory neurons (Malkinson and Spira 2010). Additional evidence that D-Asp does not activate AMPARs was the observation that CTZ did not prevent D-Asp current desensitization. CTZ has been shown to prevent desensitization at *Aplysia* sensorimotor synapses, presumably via acting at AMPARs (Antzoulatos et al. 2003). Nevertheless, L-Glu receptors in *Aplysia* referenced in the NCBI database are principally related to the AMPA and kainate subtypes, and *Aplysia* AMPA-like receptors distinct from NMDARs have been described recently (Li et al. 2009). Although our pharmacological results with CTZ, UBP302 and particularly DNQX suggest the D-AspR is not an AMPAR, D-Asp-induced current potentiation by the AMPAR-specific agents CNQX and NBQX observed here invite supposition that they may be acting as allosteric antagonists. When viewed as a whole, however, these results suggest that D-Asp likely does not activate AMPARs in *Aplysia* BSC cells.

The observed L-GluR block by bath-applied D-Asp may be either competitive inhibition or desensitization. D-Asp inhibited L-Glu-evoked currents approximately 44%, yet L-Glu did not block D-Asp-induced currents. D-Asp acting as a partial agonist of a putative NMDAR current in *Aplysia* culminated in apparent inhibition of these currents (Dale and Kandel 1993), while D-Asp directly competing with L-Glu at AMPARs without inducing current was observed in rat hippocampal neurons (Gong et al. 2005). Based on the pharmacological results presented here, it is possible that bath-applied D-Asp blocked or desensitized AMPARs, and/or a subpopulation of NMDAR-like receptors that ordinarily contribute to the whole-cell current induced by D-Asp. Interestingly, since D-Asp currents were blocked 46% by PPDA it is possible that bath-applied D-Asp blocks NMDARs containing NR2-like subunits, although NR2 subunit diversity has not been demonstrated in invertebrates (Ryan and Grant 2009).

We found support for our hypothesis that D-Asp activated a mixed collection of receptors by the summary actions of many reagents. D-Asp may activate at least two different known ion currents in *Aplysia* BSC neurons: EAATs and NMDA-like receptors containing NR2-like subunits. The component of D-Asp current that is not due to NMDA-like receptors or EAAT current may represent a unique receptor channel. It is likely that D-Asp may substitute for L-Glu in eliciting excitation at some synapses, and potentially augment L-Glu responses at synapses possessing receptors for both agonists. Studies using cocultured synaptic preparations would greatly aid in characterization of the role of these agonists. Reduced preparations would be useful in informing the behavioral relevance of D-Asp. BSC neurons have receptive fields in the buccal region that undergo sensitization (Walters et al. 2004). Facilitation of D-Asp responses by serotonin (Carlson and Fieber 2011) may play a role in this, similar to facilitation of L-Glu in other characterized systems.

Not greater than partial block of either L-Glu or D-Asp currents by L-GluR antagonists suggests that neither *Aplysia* receptor is particularly well targeted by pharmacological agents developed for use in vertebrates. Ultimately, only molecular description in cells preferentially expressing unique *Aplysia* D-Asp receptors will definitively identify the receptor. To date, however, the NR1 subunits are the sole NMDARs to have been cloned from *Aplysia* (Ha et al. 2006). This study and Carlson and Fieber (2011) strongly support the findings of Errico et al. (2011) that D-Asp is a neurotransmitter at dedicated receptors in multiple species. We have summarized the effects of the known L-Glu antagonists to support the conclusion that D-Asp activates a receptor distinct from L-GluRs.
